# Risk Factors in Predicting Prognosis of Neonatal Bacterial Meningitis—A Systematic Review

**DOI:** 10.3389/fneur.2018.00929

**Published:** 2018-11-20

**Authors:** Dan-Hua Mao, Jing-Kun Miao, Xian Zou, Na Chen, Lin-Chao Yu, Xin Lai, Meng-Yuan Qiao, Qi-Xiong Chen

**Affiliations:** ^1^Department of Neonatology, Children's Hospital, Chongqing Medical University, Chongqing, China; ^2^Chongqing International Science and Technology Cooperation base of Child Development and Critical Disorders, Chongqing, China; ^3^Ministry of Education Key Laboratory of Child Development and Disorders, Chongqing, China; ^4^Chongqing Key Laboratory of Pediatrics, Chongqing, China; ^5^Chongqing Traditional Chinese Medicine Hospital, Chongqing, China

**Keywords:** neonate, bacterial meningitis, prognostic factors, risk factors, systematic review

## Abstract

**Background:** Neonatal bacterial meningitis is a severe infection with high mortality and morbidity. It is necessary to identify factors associated with a high risk of a poor prognosis so that we can prevent them with more appropriate treatments. This study was performed to summarize the prognostic factors known to predict adverse outcomes in neonatal bacterial meningitis.

**Methods:** The Medline/PubMed, Cochrane Library and Embase databases were searched for studies of prognostic risk factors in neonates with bacterial meningitis. Studies published from the initiation of the database to April 30th, 2017 were included. The quality of cohort studies was assessed by the Newcastle-Ottawa Scale (NOS). The quality of cross-section studies was assessed by the Agency for Healthcare Research and Quality (AHRQ) scale. Each prognostic factor known to cause adverse outcomes is summarized.

**Results:** Sixteen studies were identified, including 7 cohort studies and 9 cross section studies. Seizure and high protein levels in the cerebrospinal fluid (CSF) predict a poor prognosis in this disease. Coma, the need for ventilation support, and leukopenia also had some value for predicting poor prognoses. A bulging anterior fontanelle was valuable for predicting mortality. Low CSF glucose levels, thrombocytopenia, gestational age (GA) < 37 weeks and an altered sensorium were correlated with a poor prognosis. A birth weight < 2500 g, early onset meningitis and positive CSF cultures were correlated with mortality.

**Conclusions:** This study provides a preliminary exploration of prognostic factors in neonatal bacterial meningitis and thereby fills some of the gaps in the study of prognoses in this disease. These prognostic factors can be used to predict and estimate outcomes in neonatal bacterial meningitis. Without a meta-analysis, the reliability of these factors cannot be assured. In addition, these results emphasize that there is an urgent need for a standardized protocol for follow-up and well-designed prognostic studies in neonatal bacterial meningitis.

## Introduction

Neonatal bacterial meningitis (NBM) is a serious acute infection of the central nervous system. From the day that this disease was discovered, corticosteroids, therapeutic hypothermia, monoclonal antibodies and other therapies have been used in addition to antibiotics an attempt to improve outcomes in bacterial meningitis (1, 2). However, today, mortality and morbidity remain high (approximately 10–15% and 4/1000-5/1000 of live births, respectively) globally in neonates with bacterial meningitis (3–6).

Clinical experience has revealed that some factors, such as the onset time, neonate gender, and type of pathogen, are associated with trends in the development of neonatal bacterial meningitis. Early identification of high risk factors may play a decisive role in preventing poor outcomes because it allows prompt and effective treatment. Although the guidelines of the European Society of Clinical Microbiology and Infectious Diseases (ESCMID) published in 2016 (7) revealed some of the principles of and latest developments in efforts aimed at dealing with bacterial meningitis, it did not provide any factors that significantly affect mortality or sequelae in affected neonatal patients. In addition, there is lack of systematic reviews of prognostic factors that can be used to predict poor prognoses in neonatal bacterial meningitis. Hence, this study was performed to collect the currently available evidence to systematically review the prognostic factors that predict or relate to adverse outcomes in neonate bacterial meningitis.

## Methods

The Medline/PubMed, Cochrane Library and Embase databases were systematically searched for prognostic studies that described risk factors for mortality and sequelae in neonates with bacterial meningitis. The databases were searched from the beginning of the database to April 30th, 2017. The key words used as search terms were “neonate,” “bacterial meningitis,” and “prognostic factors or outcomes.” The search strategy is listed in Appendix 1. The Open Grey, National Technical Reports Library, Gray Literature Report, and ISRCTN registry were searched for studies published as gray literature. The references cited in each of the selected studies were checked to acquire relevant studies that had not been identified by the above-described retrieval methods. All titles and abstracts were read, all relevant articles were identified, and the full text of each was obtained by two researchers separately. The final range of the articles and all correlative data or information were discussed in regular meetings, and all the data included in this article were confirmed by all authors. The following criteria were applied:

Meningitis had occurred within 0–28 days.
The results were published in English, and the full-text article could be retrieved.
Studies aimed at identifying the risk factors for a poor prognosis were defined prior to our study, and studies designed to use associative models were also included.
Cohort studies and cross-section studies in which Odds Ratios or P values for the relationship between prognostic factors and outcomes were provided were included.
Data related to tubercular or viral meningitis were excluded. Articles that did not distinguish between meningitis and sepsis were also excluded.

The quality of the cohort studies was assessed using the Newcastle-Ottawa Scale (NOS). We used 3 groups (selection, comparability, and outcomes) and 8 projects to judge their quality. The final performance on the scale was indicated by total score of “^*^,” with studies with a score of 6 or more “^*^” considered high quality. The quality of the cross section studies was assessed by the Agency for Healthcare Research and Quality (AHRQ) scale, which contains 11 items. An item was scored “0” if it was answered with “NO” or “UNCLEAR” and “1” if it was answered with “YES.” Article quality was assessed as follows: low quality = 0–3, moderate quality = 4–7, and high quality = 8–11. A figure is presented to show the risk of bias in a cohort study performed using the NOS.

When analyzing the articles, crucial points were extracted, including the author, country, year, study size, study design, analysis method, pathogen, outcome and significant prognostic factors (in a multivariate analysis with OR > 1 or *P* < 0.05 or in a univariate analysis with *p* < 0.05). These data were manually summarized. The factors evaluated in multivariate analyses without showing exact OR value or *P*-value were considered not as reliable as those included with them and were divided into univariate analyses. Outcomes were categorized under three headings: (1) mortality (2) sequelae and (3) poor outcomes (when no distinction between mortality and sequelae was drawn in the original study).

A table was formed using prognostic factors, study type, associated outcomes and their occurrences to allow readers to form their own opinions (The times of occurrences were recorded by “1x, 2x, 3x, and so on” in ascending order). Factors found to be significant in more than one study of moderate/high quality are presented and were categorized into three groups depending on study type:

Group A: cohort studies with multivariate analyses.
Group B: cohort studies with univariate analyses.
Group C: cross section studies with multivariate or univariate analyses.

To reduce the impact of bias, the prognostic factors were divided into 3 levels based on the level of evidence of the original study:

Level I factor: Showed at least “2x” in Group A.
Level II factor: Showed “1x” in Group A with at least “1x” in Group B or Group C.
Level III factor: Showed at least “1x” in each of Group B and Group C.

We established a hierarchical system in which each level had different grade (reliability) for predicting outcomes. Level I factors were at the top and were regarded as confirmed to be able to predict poor outcomes. Level II factors were considered intermediate and were considered to have some value for predicting outcomes. Level III factors were considered inferior and to show some evidence of being correlated with and potentially valuable for predicting outcomes.

## Result

### Flow path of the selection

**Figure 1:**
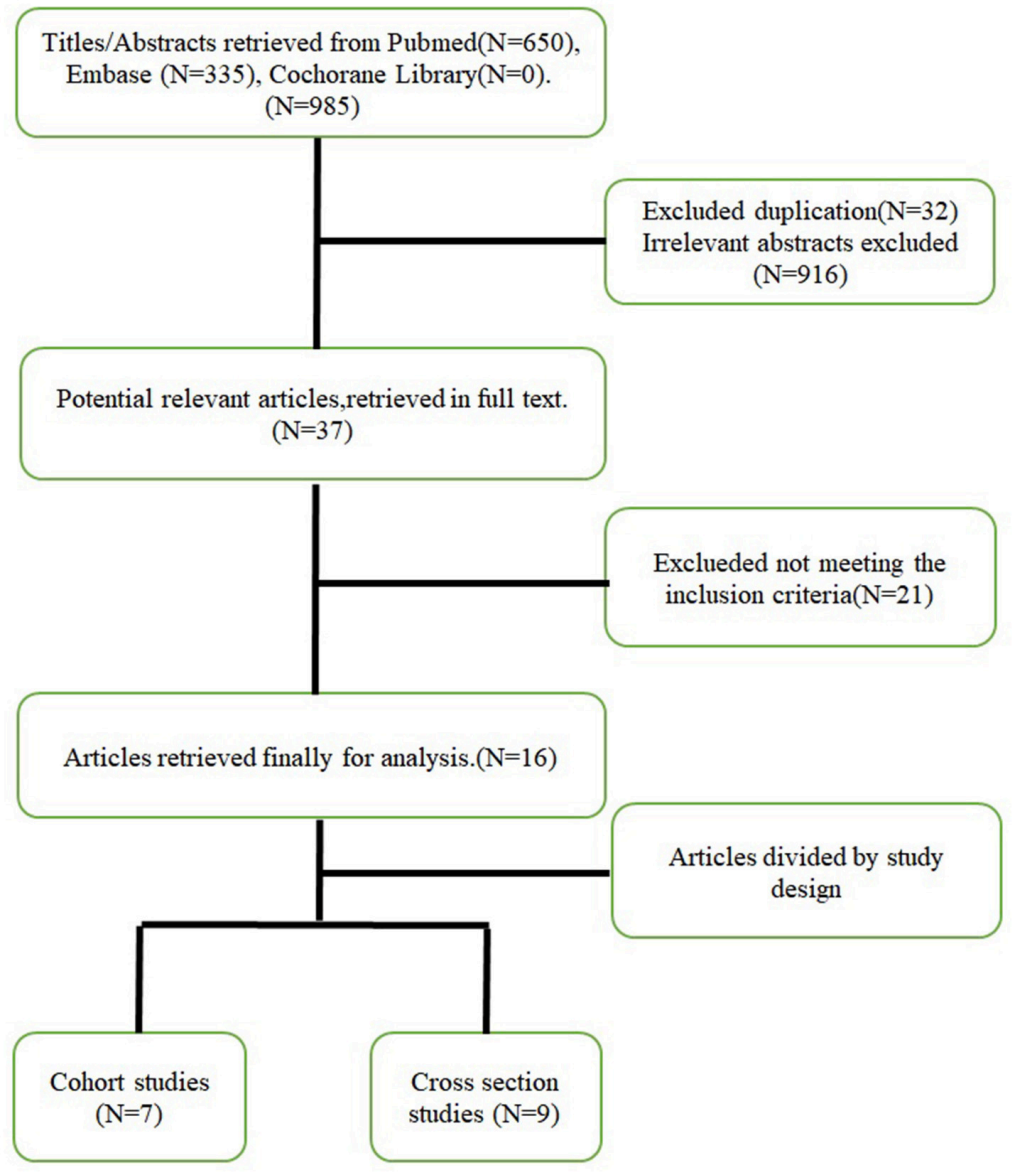
Flow path of the selection.

The flow path used for selection is shown in Figure 1. In the initial systematic search, 985 results were found, including 650 records from the PubMed/Medline database and 335 records from the Embase database. The Open Gray registry returned 3 records, and the National Technical Reports Library, Gray Literature Report, and ISRCTN registries returned 0 records. No clinical trials were found that performed a prognostic study in NBM. Of the identified records, 37 articles seemed to have high potential to meet the inclusion criteria. After we read the full text of and carefully screened each article, 21 of the articles were excluded (6 because of language, 5 because of the age of the subjects, 2 for not distinguishing between sepsis and meningitis, 3 for not running a statistical analysis of prognostic factors and outcomes, 3 for not analyzing the relationship between prognostic factors and outcomes, and 2 because we could not acquire the full-text article). Finally, 16 articles (3, 8–22) were identified. The study characteristics and quality assessments of all if the included publications are summarized in Tables 1, 2, respectively. Studies were grouped by design (cohort study or cross-sectional study) and ranked by quality.

**Table 1:** Study characteristics of included studies (cohort studies).

**Study**	**Country**	**Year**	**N**	**Study design**	**Statistical method**	**Pathogen numbers**	**Follow-up years**	**Outcome**	**Significant prognostic factors from multivariate analysis or from univariate analysis with *p* < 0.05**	**NOS scale**
Daoud et al. 9	Jordan	1992–1994	53	Prospective	Multivariate	12	8–30 m (mean 16.4 m)	Mortality and Sequela	Bulging anterior fontanelle, altered sensorium	8
Chang et al. 13	China, Taiwan	1986–2001	60	Retrospective	Multivariate	14	6 m after treatment	Mortality and Sequela	Seizure, fontanelle bulging, thrombocytopenia, CSF total protein, CSF glucose	7
Lin et al. 11	China, Taiwan	1984–2008	156 (7 lost)	Retrospective	Multivariate	19	1–2 year	Mortality and Sequela	Early onset, preterm, CSF pro > 500 mg/dl at admission, predisposing to congenital heart disease, hearing impairment, seizure	7
Klinger et al. 22	Canada	1979–1998	101	Retrospective	Multivariate	9	At 1 year of age	Mortality and Sequela	Seizure, coma, need for ventilation support, leukopenia	7
Tan et al. 12	China	2008–2014	232	Retrospective	Multivariate	11	Discharge day or at an age of 2.5–3 m	Mortality and Sequela	CSF protein, pneumonia, poor feeding, PLT	6
Kamoun et al. 14	Tunisa	1990–2012	55 (9 lost)	Retrospective	Univariate	12	1–11 year	Mortality and Sequela	Altered consciousness, CSF protein, CSF glucose	6
Bell et al. 19	Northern Ireland	1973–1986	41	Retrospective	Univariate	8	At 2 years of age or older by pediatrician	Mortality	Weight < 2,500 g, positive CSF culture	6

**Table 2:** Study characteristics of included studies (cross-section studies).

**Study**	**Country**	**Year**	**N**	**Study design**	**Statistical method**	**Pathogen types**	**Follow-up years**	**Outcome**	**Significant prognostic factors from multivariate analysis or from univariate analysis with *p* < 0.05**	**AHRQ score**
Holt et al. (15)	English	1996–1997	274	Prospective	Multivariate	Not specific	/	Mortality	Coma on admission, treated with steroids, weight < 2,000 g, GA < 33 w	9
Gaschignard et al. (3)	French	2001–2007	444	Prospective	Multivariate	Not specific	/	Mortality	Preterm	9
May et al. (8)	Australia and New Zealand	1992–2002	78	Prospective	Multivariate	Not specific	/	Mortality	Weight < 1,500 g, gram negative	8
Bortolussi et al. (18)	Australia	1968–1974	52	Retrospective	Univariate	Not specific	/	Mortality	Weight < 2,500 g, neutropenia, thrombocytopenia	8
Gebremariam et al. (10)	Ethiopia	1987–1996	55	Retrospective	Univariate	Not specific	/	Mortality	Early onset	7
Airede et al. (16)	Nigeria	1988–1990	36	Retrospective	Univariate	Not specific	/	Mortality	Weight ≤ 2,500 g, bulging anterior fontanelle, gender	7
Krebs et al. (20)	Brazil	1994–2004	87	Retrospective	Univariate	Not specific	/	Mortality	Positive CSF culture	7
Nathoo et al. (21)	Zimbabwe	1987–1988	94	Retrospective	Univariate	Not specific	/	Mortality	Weight < 2,500 g, gram negative	7
Mulder et al. (17)	Netherland	1976–1982	68	Retrospective	Univariate	GBS	/	Mortality	Weight < 2,500 g, GA < 35 w	6

### Cohort studies

Of the 16 included studies, only 7 were cohort studies. The rest (9/16) were cross section studies. Of the 7 cohort studies, 6 were retrospective studies, and the other one was a prospective study. Three of the studies were completed in China or Taiwan, China. Of the inclusion criteria, there were differences among the studies in diagnostic methods and the age of the subjects (details show in Table 3). The number of pathogen species described in the included articles varied from 8 to 19. The two main etiologic agents were found to be Group B *Streptococcus* (GBS) and *Escherichia coli* in 4 studies [not including (9, 14, 19)]. No ANOVA analyses were performed in these articles to determine the exact statistical relationship between these pathogens and outcomes. The treatment strategies used in the study projects were described in only 3 of them (show in Table 3). The follow-up time varied from 2.5 months to 11 years, and the major follow-up outcomes were sequelae and death (only one study (22) mentioned measurement outcomes on the Glasgow Coma Scale (GCS), and the time of measurement included the hospital discharge day). Another study (19) used a follow-up time of 2 years old or older in an examination performed by a pediatrician.

**Table 3:** The details of included cohort studies.

**Study**	**Diagnostic criteria**	**Treatment**	**Outcome**	**Significant prognostic factors from multivariate analysis or from univariate analysis with *p* < 0.05**
Chang et al. (13)	CSF culture positive. Leukocyte >0.1*10^∧^9/L (with predominant polymorphonuclear) or CSF protein > 1.5 g/L and CSF glucose/blood glucose < 0.5	Empiric antibiotics = ampicillin and 3rd generation cephalosporin with or without aminoglycoside	Death and severely abnormal <=> poor outcome Normal and mild abnormal <=> good outcome	Seizure, fontanelle bulging, thrombocytopenia, CSF total protein, CSF glucose
Daoud et al. (9)	CSF culture positive or blood culture positive and CSF pleocytosis > 100/mm3	Ampicillin and gentamicin (1992 to mid-1993), cefotaximin or ceftazidime combined ampicillin (mid-1993 to 1994)	Mortality Having sequela	Bulging anterior fontanelle, altered sensorium
Lin et al. (11)	CSF culture positive	Not mentioned	Mortality Death and sequela <=> poor prognosis Complete recovery <=> good prognosis	Earlyonset, preterm, CSF Pro >500 mg/dl at admission, predisposing to congenital heart disease, hearing impairment, seizure
Tan et al. (12)	Having clinical signs. Either CSF culture positive or CSF WBC > 20*10^∧^6/L and blood culture positive. GA ≥ 37 w	Not mentioned	GCS = 5 <=> good outcome GCS = 1-4 <=> bad outcome	CSF protein, pneumonia, poor feeding, PLT
Klinger et al. (22)	CSF culture positive and GA ≥ 35 w	Not mentioned	Death or moderate or severe disability <=> adverse outcome	Seizure, coma, need for ventilation support, leukopenia
Kamoun et al. (14)	CSF culture positive. The combination of leukocyte >30/mm3, CSF pro > 1.3 g/L, CSF glu < 2.2 mmol/L or CSF/blood glucose <0.4	Not mentioned	Mortality Having sequela	Altered consciousness, CSF protein, CSF glucose
Bell et al. (19)	CSF culture positive or leukocyte > 100/mm3	Aminoglycoside with benzylpicillin, chloramphenicol, or a cephalosporin	Mortality	Weight <2,500 g, positive CSF culture

### Cross-section studies

In the rest 9 cross section studies, there's large difference in study period (from 1968 to 2007). Half of the studies were run in developed countries. The major etiologic agents varied substantially from country to country and period to period. Mulder et al. (17) focused on a specific pathogen in GBS meningitis, and (8) focused on early onset meningitis (a diagnosis of early onset meningitis achieved within 48 h after delivery). The outcome in the cross section studies was mortality. The inclusion criteria were also different among the studies. In addition, half of the studies failed to mention the treatment used in NBM.

### Prognostic factors

A total of 24 factors were found to potentially predict prognoses. Some factors were not significant in any other studies, and their value could therefore not be defined. The 13 prognostic factors that were found to be significant in more than one moderate/high quality study are shown in Table 4. The prognostic factors were divided by study design and statistical method. Although 3 of the cross-section studies were prospective studies that included multivariate analyses and identified some factors that predicted prognoses, because these studies did not provide exact follow-up times, the value of these results is thought to be lower than that found in the cohort studies. Despite the fact that one study (13) included a multivariate analysis, the prognostic factors were obtained from univariate analyses (factors come from multivariate analysis were not within the definition of outcomes). In addition, some of the prognostic factors identified in Lin et al. (11) were also evaluated in a univariate analysis.

**Table 4:** Prognostic factors in neonatal bacterial meningitis.

**Prognostic factor**	**Cohort studies with multivariate analysis**			**Cohort studies with univariate analysis**			**Cross section studies with multivariate or univariate analysis**		
	**Mortality**	**Sequelae**	**Poor prognosis**	**Mortality**	**Sequelae**	**Poor prognosis**	**Mortality**	**Sequelae**	**Poor prognosis**
Early onset meningitis				1x			1x	
High CSF protein level			2x	1x		1x			1x
Low CSF glucose level					1x	1x			1x
Bulging anterior fontanelle	1x					1x	1x	
Altered sensorium		1x		1x				
Seizure			2x			1x			1x
GA < 37 w						1x	1x	
Thrombocytopenia						1x	1x	
Birth weight < 2,500 g				1x			4x	
Positive CSF culture				1x			1x	
Coma			1x				1x		1x
Need for ventilation support			1x						1x
Leukopenia			1x				1x		

### Risk of bias

**Figure 2:**
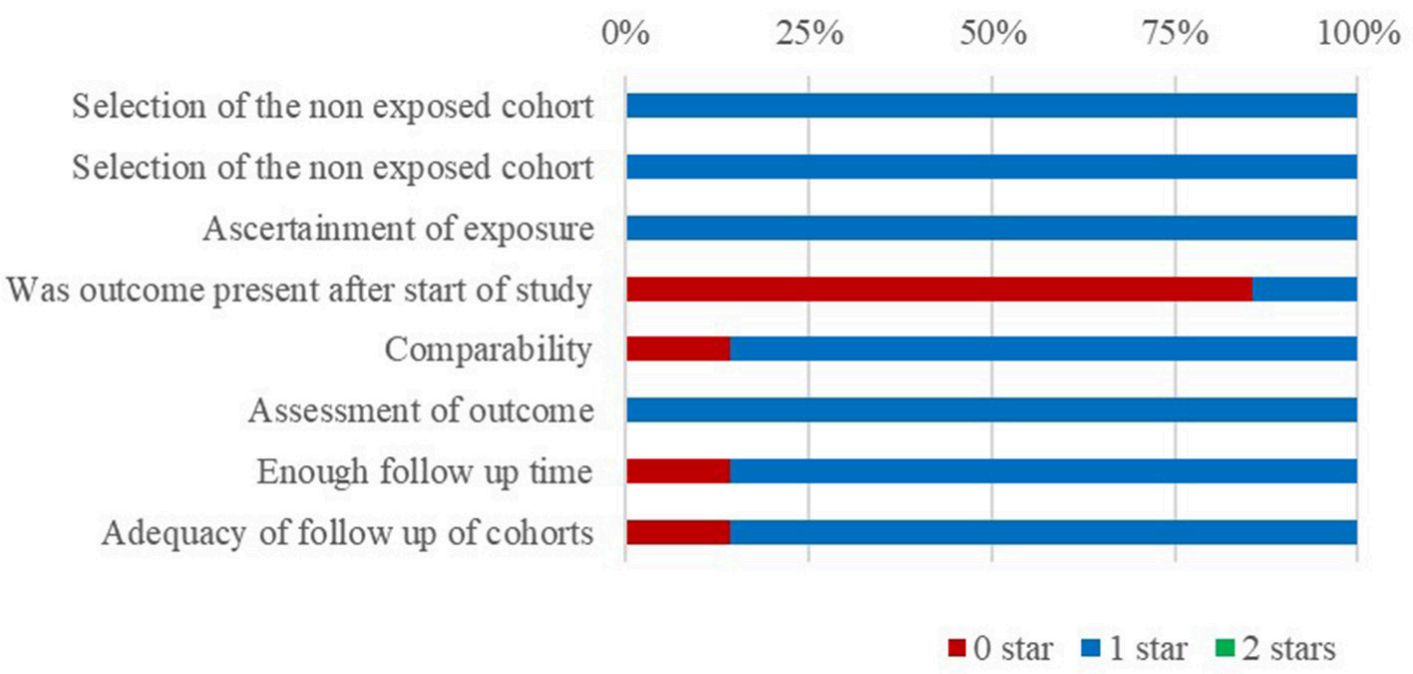
Risk of bias.

The risk of bias of the cohort studies is shown in Figure 2. All of the included studies were moderate/high quality, but only a few were high quality. Six of the included cohort studies were retrospective studies and lacked statements describing the therapy used, suggesting that there is potential for selection and comparability bias. More than half of the included cross section studies scored poor for control confounding factors, further suggesting the potential for confounder and selection bias.

### Interpretation of prognostic factors

A bulging anterior fontanelle was found to be a Level II factor with a moderate sensitivity for predicting mortality. Early onset meningitis (EOM), a birth weight < 2500 g and positive CSF culture were included as Level III factors, which were defined as significantly correlated in more than two studies (EOM was defined in two studies as bacterial meningitis that occurred within 7 days after birth).

None of the included investigations was designed to identify the relationships between certain sequela within the 13 prognostic factors. In addition, because evidence was limited, none of the factors was found to be significant for sequelae. It is important to emphasize that none of the studies reported prognostic factors specifically related to hearing loss.

Seizure and a high CSF protein level significantly predicted a poor prognosis in in more than one cohort study. These were classified as Level I factors and were mainly summarized as predictable prognostic factors. Coma, a need for ventilation support and leukopenia were identified in both multivariate and univariate analyses as Level II factors, which were considered to have some value for predicting a poor prognosis. Low CSF glucose levels, thrombocytopenia, and a GA < 37 w were Level III factors and therefore supposed to be significantly correlated with a poor prognosis. Although an altered sensorium was not defined as meeting the classification standards described above, it was accepted as a Level III factor that predicts a poor prognosis.

## Discussion

A total of 24 prognostic factors were identified in all of the included studies. Of these, 13 were found to be significant in more than one moderate/high quality study. These prognostic factors included patient characteristics, clinical manifestations and the results of laboratory examinations. Only two of the factors were classified as Level I factors, which were defined as having a high ability to predict bad outcomes. The remaining 11 factors were all found to present intermediate and low levels of evidence and will need to be tested in further multivariate analyses.

Compared to RC de Jonge et al. (23), we found that seizure, high CSF protein levels, low CSF glucose levels, leukopenia, coma and a need for ventilation support (severe respiratory distress) were significant in this research. This might be because the mechanisms underlying the development of bacterial meningitis, whether it occurs in a neonate or at any other age, are very similar: the pathogens cross the blood-brain barrier, leading to an inflammatory cascade and brain cell injury (24–26). GA < 37 w and birth weight < 2500 g are associated with prematurity in infants. In this study, while both factors were significantly correlated with outcomes, it remains unresolved whether they can be used to predict prognoses or will be eliminated when more studies are performed that remove confounding factors. The immune system is more vulnerable in more premature bodies, and this may partly explain why premature babies have such a poor prognosis. However, we wondered whether the difficulty of nursing associated with prematurity and/or another reason might also provide a part of the explanation. Additional studies are needed to clarify this question.

Thrombocytopenia was not found to predict prognoses in RC de Jonge et al. (23), but it was found to be significant in this and two other studies (27, 28). On the one hand, bacterial meningitis describes a series of immune responses, and platelets have been verified as key players in inflammation and autoimmune responses (29–31). On the other hand, sepsis may be an etiologic factor for thrombocytopenia (32), and some cases of bacterial meningitis observed in neonates are caused solely by sepsis. These two factors might partially explain why thrombocytopenia occurs in NBM. Platelet transfusion is the specific treatment for thrombocytopenia, but platelet transfusion is very likely related to mortality or any other risks and adverse events (33). The presence of thrombocytopenia indicates a severe infection, and it is necessary to give more attention to neonates with bacterial meningitis who develop thrombocytopenia and to use more positive strategies to treat the primary disease.

Diverse definitions have been used for EOM, such as occurs within 3 days vs. occurs within 7 days. Early onset sepsis (EOS) was defined as disease occurring within 3 days and may be the reason that some doctors define early onset bacterial meningitis as cases that occur within 3 days. However, others have proposed that EOM occurs within 7 days (34). In addition to this study, two of the included cohort studies and one of the included cross section studies defined EOM as bacterial meningitis occurring within 7 days after birth. The authors therefore agree with the above definition of EOM. Evidence has shown that GBS is the most common pathogen in EOS (35), and the pathogens most often observed in clinical practice in the CSF cultures of neonates with early onset bacterial meningitis are GBS. In this study, EOM was found to be a potentially predictive factor for mortality. Hence, while it is therefore reasonable to speculate that GBS infection might be associated with poor outcomes in NBM, limited evidence is available (few articles have analyzed the differences in the prognosis of patients with disease caused by different pathogens) and the positive rate in CSF culture is low. These results need to be confirmed in further studies.

A recent systematic review showed that the incidence of hearing loss was 11% (range, 2–35%) and that 5% of patients experienced profound hearing loss after having childhood bacterial meningitis (36). In addition, some other studies showed that seizures, serum C-reactive protein levels, and disease severity were risk factors for hearing impairment (37, 38) after childhood bacterial meningitis. However, previous articles have rarely analyzed its incidence or factors that independently predict hearing loss in neonates with bacterial meningitis. Even though the studies included in this study had reported prognostic factors for sequelae, hearing loss was not separately analyzed. Another study found that some cases with normal hearing or moderate hearing loss later became severely impaired after exhibiting bacterial meningitis (39). These results indicate that late-onset hearing loss should not be underestimated in NBM. Currently, Universal Newborn Hearing Screening (UNHS) is performed worldwide and has allowed great progress to be made in fighting hearing loss. However, in neonates with bacterial meningitis, the timing of the first screening, the follow-up timing used in patients with normal results at the first screening (e.g., those with late-onset hearing loss), and the frequency of follow-up after NBM remain unclear despite the fact that the Joint Committee on Infant Hearing (JCIH) updated the guidelines for detection and intervention programs for hearing loss in 2007 (40) and further supplemented them in 2013 (41). Since we know that early identification and intervention result in significantly better outcomes, we sought to determine what we can do to reduce hearing loss and help babies recover, and we concluded that special guidelines are needed for early identification and intervention to prevent hearing loss after NBM.

CSF protein and glucose levels were found to be prognostic factors in this study. In addition, CSF protein and glucose levels are part of the diagnostic criteria used to identify bacterial meningitis. While Tan et al. (12) performed an ROC curve analysis of CSF protein levels and found that the best cutoff for predicting outcomes was 1880 mg/L, because no one else has defined a best cutoff point, the specific numerical values for CSF protein and glucose levels that indicate a poor outcome remain uncertain. More studies of the cerebrospinal fluid (CSF) manifestations of NBM and their correlations with prognoses are needed.

Although one large meta-analysis showed that corticosteroids significantly reduced hearing loss and neurological sequelae in children and adults (42), another recent study performed in neonates included two randomized controlled trials and suggested that the capacity of steroids to reduce death and hearing loss (1) remains equivocal. One of the studies (15) included in this report also proposed that using steroids was significantly associated with poor prognoses. This shows that the value of administering steroids in neonatal bacterial meningitis remains uncertain despite its clear value in children and adults, and steroids should therefore be used with special caution in this population.

The study of the relationship between prognoses and risk factors was limited by the heterogeneity observed among the included studies in diagnostic criteria, treatment and follow-up. Thus, only a systematic review was performed here. Without a meta-analysis, the reliability of these factors cannot be assured. In addition, our search for guidelines for bacterial meningitis revealed that there are few such guidelines specific for neonates. As time goes on, therapies for neonatal bacterial meningitis have progressively advanced. However, there is currently a lack of systematic and standard protocols for therapy and follow-up in this population. In addition, obtaining a deeper understanding of the relationships between risk factors and prognoses in neonatal bacterial meningitis will require additional well-designed prognostic studies and quantitative analyses.

## Limitation

In the process of article selection, 6 articles not written in English and 2 articles for which the full-text could not be obtained were excluded; however, the abstracts of these studies mentioned a few prognostic factors with significant value for predicting outcomes, and this may have caused publication bias. In addition, when screening all the potentially relevant articles, the title and abstract were used as a first-step selection criterion, and those very few articles that may not have described their research very clearly in the abstract may have been eliminated in this step.

Because of restrictions on the different measurements used to evaluate outcomes in each article, a very catch-all term, “poor prognosis” (as previously stated), was used to define undesirable outcomes in neonatal bacterial meningitis; this may have led readers to be confused about the specific prognosis of this disease. In addition, because the quantity of included articles was small, a subgroup analysis could not be run to explore the impact of diagnoses, treatments, and follow-up times on prognosis, and this also caused heterogeneity.

Because there was heterogeneity in each study, a systematic review but not a meta-analysis was performed to obtain results for prognostic factors in NBM. It is clear that the statistical effect of qualitative analyses is far inferior to that of quantitative analysis, and this research can therefore provide only a preliminary exploration of evidence-based medicine. The quality assessment showed that the included studies may have presented a risk of selection and confounder bias. Thus, it is hard to estimate the statistical effect of these data on prognostic factors in a systematic review, and only one interpretation of the results is provided here. Every reader should draw their own conclusions.

## Conclusion

This study provides a preliminary exploration of prognostic factors in neonatal bacterial meningitis. This fills in some of the gaps in the study of prognoses in this disease. Existing studies contain a great deal of heterogeneity related to the diagnosis, treatment, and follow-up of this disease, and this largely restricts the study of relationships between prognoses and risk factors. Without a meta-analysis, the reliability of these factors as prognostic indicators cannot be assured. In addition, this conclusion emphasizes the urgent need for a standard follow-up protocol and well-designed prognostic studies in neonatal bacterial meningitis.

## Author contributions

D-HM conceptualized and designed the study, drafted the initial manuscript, and approved the final manuscript as submitted. J-KM carried out the initial analyses, reviewed and revised the manuscript, and approved the final manuscript as submitted. XZ and NC helped with the selection of articles. L-CY and XL helped with the construction of article. M-YQ given crucial advice in statistical process. Q-XC designed the study, reviewed and revised the manuscript, and approved the final manuscript as submitted. All authors approved the final manuscript as submitted and agree to be accountable for all aspects of the work.

### Conflict of interest statement

The authors declare that the research was conducted in the absence of any commercial or financial relationships that could be construed as a potential conflict of interest.

## References

[1] Ogunlesi TA Odigwe CC Adjuvant corticosteroids for reducing death in neonatal bacterial meningitis. Cochrane Datab Sys Rev. (2015) 11:CD010435 10.1002/14651858.CD010435.pub2 PMC10542916 26560739

[2] Nau R Djukic M Spreer A Eiffert H. Bacterial meningitis: new therapeutic approaches. Expert Rev Anti Infect Ther. (2013) 11:1079–95. 10.1586/14787210.2013.839381 24073921

[3] Gaschignard J Levy C Romain O Cohen R Bingen E Aujard Y. Neonatal bacterial meningitis: 444 cases in 7 years. Pediatric Infectious Disease Journal. (2011) 30:212–7. 10.1097/INF.0b013e3181fab1e7 21416693

[4] Softić I Tahirović H HasanhodžIć M. Neonatal bacterial meningitis: results from a cross-sectional hospital based study. Acta Med Acad. (2015) 44:117–23. 10.5644/ama2006-124.139. 26702907

[5] Baud O Aujard Y. Neonatal bacterial meningitis. Handb Clin Neurol. (2013) 112:1109-13. 10.1016/B978-0-444-52910-7.00030-1 23622318

[6] Ben HH Ben HKA Hamza MA Ayadi A Soua H Khedher M [clinical outcome and prognosis of neonatal bacterial meningitis]. Arch Pediatr. (2013) 20:938–44. 10.1016/j.arcped.2013.05.005 23829970

[7] van de Beek D Cabellos C Dzupova O Esposito S Klein M Kloek AT Escmid guideline: diagnosis and treatment of acute bacterial meningitis. Clin Microbiol Infect. (2016) 22(Suppl 3):S37 10.1016/j.cmi.2016.01.007 27062097

[8] May M Daley AJ Donath S Isaacs D. Early onset neonatal meningitis in australia and new zealand, 1992-2002. Archiv Dis Childh Fetal Neonatal Edn. (2005) 90:F324. 10.1136/adc.2004.066134 15878934 PMC1721922

[9] Daoud AS Alsheyyab M Abuekteish F Obeidat A Ali AA Elshanti H. Neonatal meningitis in northern jordan. J Tropic Pediatr. (1996) 42:267. 10.1093/tropej/42.5.267 8936956

[10] Gebremariam A. Neonatal meningitis in addis ababa: a 10-year review. Ann Tropic Paediatr. (1998) 18:279–83. 10.1080/02724936.1998.11747960 9924582

[11] Lin MC Chi H Chiu NC Huang FY Ho CS. Factors for poor prognosis of neonatal bacterial meningitis in a medical center in northern Taiwan. J Microbiol Immunol. (2012) 45:442–7. 10.1016/j.jmii.2011.12.034 22571998

[12] Tan J Kan J Qiu G Zhao D Ren F Luo Z. Clinical prognosis in neonatal bacterial meningitis: the role of cerebrospinal fluid protein. PLoS ONE (2015) 10:e0141620. 10.1371/journal.pone.0141620 26509880 PMC4625018

[13] Chang CJ Chang WN Huang LT Huang SC Chang YC Hung PL. Neonatal bacterial meningitis in southern taiwan. Pediat Neurol. (2003) 29:288–94. 10.1016/S0887-8994(03)00273-X 14643389

[14] Kamoun F Dowlut MB Ameur SB Sfaihi L Mezghani S Chabchoub I. Neonatal purulent meningitis in southern tunisia: epidemiology, bacteriology, risk factors and prognosis. Fetal Pediat Pathol. (2015) 34:233. 10.3109/15513815.2015.1051252 26083897

[15] Holt DE Halket S De LJ Harvey D. Neonatal meningitis in england and wales: 10 years on. Archiv Dis Childh Fetal Neonatal Edn. (2001) 84:85–9. 10.1136/fn.84.2.F85 11207221 PMC1721232

[16] Airede AI. Neonatal bacterial meningitis in the middle belt of nigeria. Develop Med Child Neurol. (1993) 35:424–30. 10.1111/j.1469-8749.1993.tb11664.x 8495823

[17] Mulder CJ Zanen HC. Neonatal group b streptococcal meningitis. Archiv Dis Childh. (1984) 59:439–43. 10.1136/adc.59.5.439 6375583 PMC1628518

[18] Bortolussi R Krishnan C Armstrong D Tovichayathamrong P. Prognosis for survival in neonatal meningitis: clinical and pathologic review of 52 cases. Can Med Assoc J. (1978) 118:165–8. 757384 PMC1880370

[19] Bell AH Brown D Halliday HL Mcclure G Mcreid M. Meningitis in the newborn–a 14 year review. Arch Dis Childh. (1989) 64:873. 10.1136/adc.64.6.873 2774622 PMC1792587

[20] Krebs VL Costa GA. Clinical outcome of neonatal bacterial meningitis according to birth weight. Arq Neuropsiquiatr. (2007) 65:1149–53. 10.1590/S0004-282X2007000700011 18345420

[21] Nathoo KJ Pazvakavamba I Chidede OS Chirisa C. Neonatal meningitis in harare, zimbabwe: a 2-year review. Ann Tropic Paediatr. (1991) 11:11–5. 10.1080/02724936.1991.11747472 1714689

[22] Klinger G Chin CN Beyene J Perlman M. Predicting the outcome of neonatal bacterial meningitis. Pediatrics (2000) 106:477. 10.1542/peds.106.3.477 10969090

[23] de Jonge RC van Furth AM Wassenaar M Gemke RJ Terwee CB Predicting sequelae and death after bacterial meningitis in childhood: a systematic review of prognostic studies. BMC Infect Dis. (2010) 10:232 10.1186/1471-2334-10-232 20684796 PMC2921388

[24] Barichello T Fagundes GD Generoso JS Elias SG Simões LR Teixeira AL. Pathophysiology of neonatal acute bacterial meningitis. J Med Microbiol. (2013) 62(Pt 12):1781. 10.1099/jmm.0.059840-0 23946474

[25] Putz K Hayani K Zar FA. Meningitis. Primary Care Clin Off Pract. (2013) 40:707. 10.1016/j.pop.2013.06.001 23958365

[26] Heckenberg SGB Brouwer MC Beek DVD. Bacterial meningitis. Handb Clin Neurol. (2014) 121:1361–75. 10.1016/B978-0-7020-4088-7.00093-6 24365425

[27] De FMAMM Bezerra PC Guedes DL Cabral DB De BMD Prognostic indicators in bacterial meningitis: a case-control study. Braz J Infect Dis. (2013) 17:538–44. 10.1016/j.bjid.2013.01.016 23835007 PMC9425123

[28] van de Beek D De GJ Spanjaard L Weisfelt M Reitsma JB Vermeulen M Clinical features and prognostic factors in adults with bacterial meningitis. N Engl J Med. (2005) 351:1849 10.1056/NEJMoa040845 15509818

[29] Jenne CN Kubes P. Platelets in inflammation and infection. Platelets (2015) 26:286. 10.3109/09537104.2015.1010441 25806786

[30] Thomas MR Storey RF. The role of platelets in inflammation. Thromb Haemost. (2015) 114:449–58. 10.1160/TH14-12-1067 26293514

[31] Herter JM Rossaint J Zarbock A. Platelets in inflammation and immunity. J Thromb Haemost. (2015) 12:1764–75. 10.1111/jth.12730 25224706

[32] Ulusoy E Özlem T Duman N Kumral A Irken G Ören H. Thrombocytopenia in neonates: causes and outcomes. Ann Hematol. (2013) 92:961–7. 10.1007/s00277-013-1726-0 23519382

[33] Gunnink SF Vlug R Fijnvandraat K Jg VDB Stanworth SJ Lopriore E. Neonatal thrombocytopenia: etiology, management and outcome. Expert Rev Hematol. (2014) 7:387. 10.1586/17474086.2014.902301 24665958

[34] Sinha IP Nelson textbook of pediatrics. Semin Fetal Neonatal Med. (2012) 17:380 10.1016/j.siny.2012.09.006

[35] Simonsen KA Andersonberry AL Delair SF Davies HD. Early-onset neonatal sepsis. Clin Microbiol. Rev (2014) 27:21–47. 10.1128/CMR.00031-13 24396135 PMC3910904

[36] Rodenburg-Vlot MB Ruytjens L Oostenbrink R Goedegebure A Mp VDS. Systematic review: incidence and course of hearing loss caused by bacterial meningitis: in search of an optimal timed audiological follow-up. Otol Neurotol. (2016) 37:1–8. 10.1097/MAO.0000000000000922 26649601

[37] Adachi N Ito K Sakata H. Risk factors for hearing loss after pediatric meningitis in japan. Ann Otol Rhinol Laryngol. (2010) 119:294–6. 10.1177/000348941011900504 20524573

[38] Roine I Pelkonen T Cruzeiro ML Kataja M Peltola H Pitkäranta A. Hearing impairment and its predictors in childhood bacterial meningitis in angola. Pediatr Infect Dis J. (2013) 32:563. 10.1097/INF.0b013e3182880037 23411625

[39] Roine I Pelkonen T Cruzeiro ML Kataja M Aarnisalo A Peltola H. Fluctuation in hearing thresholds during recovery from childhood bacterial meningitis. Pediatr Infect Dis J. (2014) 33:253. 10.1097/INF.0000000000000218 24569385

[40] American Academy of Pediatrics Joint Committee on Infant Hearing Year 2007 Position Statement: principles and guideline for early hearing detection and intervention programs. Pediatrics (2007) 120:898–921. 10.1542/peds.2007-2333 17908777

[41] Joint Committee on Infant Hearing Muse C Harrison J Yoshinaga-Itano C Grimes A Brookhouser PE Supplement to the JCIH 2007 position statement: principles and guidelines for early intervention following confirmation that a child is deaf or hard of hearing. Pediatrics (2013) 131:e1324–49. 10.1542/peds.2013-0008 23530178

[42] Beek DVD Brouwer MC Mcintyre P Prasad K Corticosteroids for acute bacterial meningitis. Cochrane Datab Syst Rev. (2015) 12:CD004405 10.1002/14651858.CD004405.pub PMC6491272 26362566

