# Corrigendum: Risk Factors in Predicting Prognosis of Neonatal Bacterial Meningitis—A Systematic Review

**DOI:** 10.3389/fneur.2019.01119

**Published:** 2019-10-22

**Authors:** Dan-Hua Mao, Jing-Kun Miao, Xian Zou, Na Chen, Lin-Chao Yu, Xin Lai, Meng-Yuan Qiao, Qi-Xiong Chen

**Affiliations:** ^1^Department of Neonatology, Children's Hospital, Chongqing Medical University, Chongqing, China; ^2^Chongqing International Science and Technology Cooperation base of Child Development and Critical Disorders, Chongqing, China; ^3^Ministry of Education Key Laboratory of Child Development and Disorders, Chongqing, China; ^4^Chongqing Key Laboratory of Pediatrics, Chongqing, China; ^5^Chongqing Traditional Chinese Medicine Hospital, Chongqing, China

**Keywords:** neonate, bacterial meningitis, prognostic factors, risk factors, systematic review

In the published article, there was an error regarding the affiliation for “Qi-Xiong Chen.” As well as having affiliation(s) 5, they should also have the “Department of Neonatology, Children's Hospital, Chongqing Medical University, Chongqing, China.”

The authors apologize for this error and state that this does not change the scientific conclusions of the article in any way. The original article has been updated.

