# Supplementary material for: Risk Factors in Predicting Prognosis of Neonatal Bacterial Meningitis—A Systematic Review
Source: Front Neurol. 2018 Nov 20;9:929. doi: 10.3389/fneur.2018.00929 (PMC6255960; doi:10.3389/fneur.2018.00929)
Supplement: Supplementary file 1 [file Data_Sheet_1.docx]

**Appendix 1**

**Search strategy:**

**PubMed**: ((Meningitides, Bacterial[Title/Abstract] OR purulent meningitis[Title/Abstract] OR suppurative meningitis[Title/Abstract] OR Bacterial Meningitides[Title/Abstract] OR Bacterial Meningitis)[Title/Abstract] AND (Infants, Newborn[Title/Abstract] OR Newborn[Title/Abstract] OR Infant[Title/Abstract] OR Newborn Infants[Title/Abstract] OR Newborns[Title/Abstract] OR Newborn[Title/Abstract] OR Neonate[Title/Abstract] OR Neonates[Title/Abstract] OR Neonatal)[Title/Abstract] AND (Prognoses[Title/Abstract] OR Prognostic Factors[Title/Abstract] OR Factor, Prognostic[Title/Abstract] OR Factors, Prognostic[Title/Abstract] OR Prognostic Factor[Title/Abstract] OR outcome[Title/Abstract] OR follow up[Title/Abstract] OR follow-up)[Title/Abstract])

**Embase**: 'bacterial meningitis'/exp OR purulent AND meningitis OR suppurative AND meningitis AND ('newborn'/exp OR neonatal AND 'infant'/exp OR infants OR newborns OR 'neonate'/exp OR neonates) AND (prognoses OR prognostic AND factors OR prognostic AND factor OR prognosis OR 'outcome'/exp OR 'clinical outcome'/exp OR 'follow up'/exp)
